# Internet Use Frequency and Patient-Centered Care: Measuring Patient Preferences for Participation Using the Health Information Wants Questionnaire

**DOI:** 10.2196/jmir.2615

**Published:** 2013-07-01

**Authors:** Bo Xie, Mo Wang, Robert Feldman, Le Zhou

**Affiliations:** ^1^School of Nursing & School of InformationUniversity of Texas at AustinAustin, TXUnited States; ^2^Department of ManagementUniversity of FloridaGainesville, FLUnited States; ^3^Department of Behavioral and Community HealthUniversity of MarylandCollege Park, MDUnited States

**Keywords:** patient-centered care, patient preference, shared decision-making, patient participation, Internet

## Abstract

**Background:**

The Internet is bringing fundamental changes to medical practice through improved access to health information and participation in decision making. However, patient preferences for participation in health care vary greatly. Promoting patient-centered health care requires an understanding of the relationship between Internet use and a broader range of preferences for participation than previously measured.

**Objective:**

To explore (1) whether there is a significant relationship between Internet use frequency and patients’ overall preferences for obtaining health information and decision-making autonomy, and (2) whether the relationships between Internet use frequency and information and decision-making preferences differ with respect to different aspects of health conditions.

**Methods:**

The Health Information Wants Questionnaire (HIWQ) was administered to gather data about patients’ preferences for the (1) amount of information desired about different aspects of a health condition, and (2) level of decision-making autonomy desired across those same aspects.

**Results:**

The study sample included 438 individuals: 226 undergraduates (mean age 20; SD 2.15) and 212 community-dwelling older adults (mean age 72; SD 9.00). A significant difference was found between the younger and older age groups’ Internet use frequencies, with the younger age group having significantly more frequent Internet use than the older age group (younger age group mean 5.98, SD 0.33; older age group mean 3.50, SD 2.00; *t*_436_=17.42, *P*<.01). Internet use frequency was positively related to the overall preference rating (*γ*=.15, *P*<.05), suggesting that frequent Internet users preferred significantly more information and decision making than infrequent Internet users. The relationships between Internet use frequency and different types of preferences varied: compared with infrequent Internet users, frequent Internet users preferred *more* information but *less* decision making for diagnosis (*γ*=.57, *P*<.01); *more* information and *more* decision-making autonomy for laboratory test (*γ*=.15, *P*<.05), complementary and alternative medicine (*γ*=.32, *P*<.01), and self-care (*γ*=.15, *P*<.05); and *less* information but *more* decision-making autonomy for the psychosocial (*γ*=-.51, *P*<.01) and health care provider (*γ*=-.27, *P*<.05) aspects. No significant difference was found between frequent and infrequent Internet users in their preferences for treatment information and decision making.

**Conclusions:**

Internet use frequency has a positive relationship with the overall preferences for obtaining health information and decision-making autonomy, but its relationship with different types of preferences varies. These findings have important implications for medical practice.

## Introduction

Patient participation in health care decision-making has both legal and ethical grounds [1]. It is increasingly recognized as a cornerstone of patient-centered health care that can improve health care quality and outcomes [2-4] and reduce utilization of health care resources [5] and costs [6,7]. The recent shift from a paternalistic to a shared or informed model of health care decision-making [8-14] has drawn much attention to patient participation, although there is little consensus regarding exactly what patient participation entails [15]. Patient preferences or desire for the amount of information about different aspects of a health condition and level of decision-making autonomy across different aspects of a health condition are commonly used as two major indicators of patient participation, eg, [11,12,16]. However, these two types of preferences are often measured differently across studies [17], making it difficult to compare reported findings.

### The Breadth of Patient Preferences for Participation

The instruments commonly used for measuring patient preferences, established well before the prevalence of Internet use in contemporary health care, focus on a limited range of types of health information and an even more limited range of types of decision making. All of the commonly used instruments measuring preferences for obtaining health information include measures of preferences for obtaining information about treatment and diagnosis [16,18-24]. Several also include measures of preferences for obtaining information about laboratory testing/medical examination [16,18,21-24] and physical/self-care [20,22-24], but only two include measures of preference for obtaining psychosocial information [20,22]. Meanwhile, the instruments commonly used for measuring preferences for decision-making autonomy all measure primarily or even exclusively preference for participation in (standard) *treatment* decision making [16,18,19,23,25]. Other types of decision making, such as decisions regarding what medical facility to use or whether to seek complementary or alternative treatments, are understudied or even completely missing from these widely used instruments.

Currently, there is no known validated instrument measuring preferences for obtaining online health information or decision-making autonomy based on the information obtained online. However, Internet studies have found a broader range of preferences for obtaining health information and decision making autonomy than found in earlier studies [26-32]. For instance, while information about diagnosis and treatment still comprises the main types of health information that older adults seek online, several other types of health information (eg, information about nutrition, exercise, and body weight; health care providers; and alternative treatments) are also commonly sought online by older Internet users [33]. Also, using information obtained online, individuals are making a wide range of decisions regarding, eg, treatment, health care facilities and providers, how to interact with physicians (eg, what questions to ask and how to ask during an office visit), how to cope with a condition, and how to think about healthy eating, exercise, or stress management [34-38]. Some Internet studies have even revealed Internet users making decisions regarding diagnosis based on the information they obtained online [28,39].

This new broader coverage of the types of health information and decision making has helped to reveal interesting phenomena previously understudied or ignored. It also calls for a more systematic examination of the relationship between Internet use and a broad range of information and decision-making preferences.

### Measuring Preferences for Participation: The Health Information Wants Questionnaire

Derived from a grounded theory study, our health information wants (HIW) framework encompasses a broad range of types of information and decision making and presents each type of information as corresponding to one type of decision making [40]. Building on and further testing the HIW framework, we developed the Health Information Wants Questionnaire (HIWQ) through a multistage process over the course of 2 years [17,41]. The HIWQ differs from prior instruments in at least three important ways. First, it measures preferences for seven types of health information and decision making—information and decision making about diagnosis, treatment, laboratory testing, self-care, complementary and alternative medicine (CAM), psychosocial aspect, and health care providers. Second, the items on the information dimension parallel those on the decision-making dimension (ie, each item on the Information Scale has a corresponding, parallel item on the Decision Making Scale), making it possible to more directly compare preferences for participation in different types of information seeking and decision making. Finally, the HIWQ has a built-in consideration for exploring potential impacts of Internet use frequency on preferences for obtaining health information and decision-making autonomy. Detailed descriptions of the development process of the HIWQ, including our rationale for focusing on these seven types of information/decision making and the selection and development of the specific items within each type, are reported elsewhere [17,42].

In this paper, we report findings from the first large sample study using the HIWQ, focusing specifically on the relationship between *Internet use frequency* and preferences among undergraduate students and older adults. We selected these two particular age groups mainly because of the sharp contrast between their Internet use frequencies: the younger age group typically has the highest level of Internet use frequency, whereas the older adult age group has the lowest [43]. Findings from the same large sample study focusing on the relationship between age and each type of preference are reported elsewhere [42].

### Research Questions

Previous research has suggested that factors such as age, gender, education, culture, the role of being a patient, severity of health condition, and personality are related to patients’ preferences for participation in their own health care [16,19,21,44-50]. Given the accumulating amount of evidence in the literature suggesting connections between Internet use and patient participation, we asked the following primary research question (RQ):

RQ1: Is there a significant relationship between Internet use frequency and the overall preferences for obtaining health information and decision-making autonomy?

Previous research has indicated that preferences for participation are highly variable [51-55]. However, to date there is little knowledge about how different Internet users may have different preferences for participation. Recognizing this gap in the literature, we asked another primary RQ:

RQ2: Does the relationship between Internet use frequency and information and decision-making preferences differ with respect to seven different aspects of health conditions—diagnosis, treatment, laboratory testing, self-care, CAM, psychosocial aspect, and health care providers?

## Methods

### Participants

A convenience sample of 438 individuals participated in this study. Participants included 226 undergraduate students majoring in a variety of disciplines at a large state university and 212 older adults recruited from senior-oriented computer classes held at public libraries and senior centers. Participants were recruited through flyers posted in building hallways and message boards, advertisements in local newspapers, and word of mouth. Demographic characteristics of the participants are reported in Table 1 (following the Health and Retirement Study [56], we coded eight conditions—high blood pressure, diabetes, cancer, lung disease, heart disease, stroke, psychiatric problems, and arthritis—as “major” health conditions and all other conditions as “minor” health conditions).

### Materials

The data reported here were obtained using the 21-item HIWQ. This 21-item instrument is a psychometrically improved version (in terms of both reliability and construct validity) of our original 40-item HIWQ [41]. In addition, it significantly shortens the time required by participants to complete it. This self-administered instrument includes two main scales: the Information Preference Scale and the Decision Making Preference Scale. Each of these scales contains seven subscales with parallel items in the following information and decision-making categories: diagnosis (items 1-4), treatment (items 5-7), laboratory testing (items 8-10), self-care (items 11-13), CAM (items 14-16), psychosocial aspect (items 17-19), and health care providers (items 20-21) in the information and decision-making subscales (Multimedia Appendix 1).

On the Information Preference Scale, participants indicated their preferences for each type of information (eg, How much information would you like to have about how severe a health condition is) on a 5-point Likert-type scale, in which response choices ranged from 1 (None) to 5 (All). On the Decision Making Preference Scale, participants also indicated their preferences for each type of health decision making on a 5-point Likert-type scale (eg, Who do you think should make the decision regarding how severe a health condition is). Adapted from Ende et al [16], response choices were the doctor alone (1), mostly the doctor (2), the doctor and myself equally (3), mostly myself (4), and myself alone (5).

In addition to the 21 parallel items on the Information and Decision Making Scales, the HIWQ also included items measuring age (younger vs older), gender (male vs female), general health status, health condition (major vs minor), whether the condition was current or past, how long the condition lasted, severity of the condition, how knowledgeable the participant was about the condition, marital status, education level, ethnicity, income level, and Big Five personality (extraversion, agreeableness, conscientiousness, neuroticism, and openness). As summarized in several review articles [51-55], these variables were found to be related to preferences for obtaining health information and decision-making autonomy. These variables were therefore used as control variables in all relevant analyses reported here.

Before completing the Information and Decision Making Scales, participants were asked to first think about a specific health condition that they had in the past or currently had and to continue thinking about this specific health condition while filling out the rest of the questionnaire.

### Procedure

Completion of the instrument took place in a quiet university classroom or office for the undergraduate participants and in a quiet meeting room in a public library or senior center for the older participants. Prior to data collection, all participants completed the informed consent form, approved by the Institutional Review Board of the authors’ university. Participants were instructed to complete the instrument independently, using paper and pen. On average, it took approximately 15-25 minutes for an undergraduate participant and 30-45 minutes for an older adult to complete the instrument. Data collection took place from May to December 2010.

### Data Analysis

Data in the current study had a nested structure in which each participant rated items in two dimensions (ie, information preference and decision-making preference). The subscale and overall dimension scores were first calculated as means across relevant items. Following the strategy used by Ende et al [16], these original scores were then rescaled to have a midpoint of 50 and ranges from 0 (corresponding to least desire for information seeking or decision making) to 100 (corresponding to strongest desire for information seeking or decision making). The rescaling was done by linearly transforming the original score, ie, rescaled score=(raw score-1)*25. This rescaling strategy allowed us to compare the scores of the information and decision-making dimensions. Internet use frequency was between-subject level (ie, Level 2) predictor whereas dimension of preference ratings was a within-subject level predictor (ie, Level 1). Preference rating was the outcome variable. Since Internet use frequency is a continuous variable, repeated-measure ANOVA is not appropriate for testing its interaction effect with rating dimension on preference ratings. Therefore, we used the multilevel modeling technique [57] to estimate the interaction effect of Internet use frequency and rating dimension on preference ratings. Dimension of preference was coded as a dummy variable with decision-making preference = “0” and information preference = “1”, which had a random effect on preference ratings. Internet use frequency was treated as Level-2 predictor, which had effects on the random intercept of preference ratings and on the random slope of the dimension-rating relationship. In addition, we controlled for the main effects of age group, gender, general health status, whether had health condition in the past or current, how long had the condition, severity of the condition, knowledge of the condition, marital status, education, ethnicity, income, and Big Five personalities on preference ratings in the model. (Gender was coded as 1=male and 0=female. Health condition was coded as 1=major and 0=minor. Condition time was coded as 1=current and 0=past. Marital status was coded by dummy coding scheme, with married as the referent group. Ethnicity was coded by dummy coding scheme, with white as the referent group.)

**Table 1:** Demographic characteristics of study participants.

Variable		Young n=226	Older n=212	Total n=438
**Age**				
	Minimum	18	50	18
	Maximum	32	100	100
	Mean	20.31	71.92	44.16
	SD	2.15	9.00	26.52
**Gender, n (%)**				
	Female	165 (73.0)	139 (65.6)	304 (69.4)
	Male	61 (27.0)	73 (34.4)	134 (30.6)
**Marriage status, n (%)**				
	Married	2 (.9)	72 (34.0)	74 (16.9)
	Single	217 (96.0)	30 (14.1)	247 (56.4)
	Separated	2 (.9)	4 (2.0)	6 (1.4)
	Divorced	1 (.4)	32 (15.1)	33 (7.5)
	Widowed	3 (1.3)	74 (34.7)	77 (17.6)
	Living as married	1 (.4)	0 (0)	1 (.2)
**Highest level of education, n (%)**				
	Less than high school graduate	0 (0)	9 (4.2)	9 (2.1)
	High school graduate/GED	72 (31.9)	63 (29.7)	135 (30.8)
	Vocational training	1 (.4)	13 (6.1)	14 (3.2)
	Some college/associate’s degree	135 (59.7)	56 (26.4)	191 (43.6)
	Bachelor’s degree	17 (7.5)	35 (16.5)	52 (11.9)
	Master’s degree or other postgraduate training	1 (.4)	30 (14.2)	31 (7.1)
	Doctoral degree	0 (0)	6 (2.8)	6 (1.4)
**Membership in ethnic group, n (%)**				
	Asian	22 (9.7)	11 (5.2)	33 (7.5)
	African American	117 (51.8)	105 (49.5)	222 (50.7)
	Latino/Hispanic	8 (3.5)	8 (3.8)	16 (3.7)
	Native American/American Indians/Alaska Native	1 (0.4)	2 (0.9)	3 (0.7)
	Native Hawaiian/Pacific Islander	0 (0)	2 (0.9)	2 (0.5)
	White	78 (34.5)	84 (39.6)	162 (37.0)
**Annual household income, n (%)**				
	Less than $20,000	56 (24.8)	45 (21.2)	101 (23.1)
	$20,000-$29,999	7 (3.1)	31 (14.6)	38 (8.7)
	$30,000-$39,999	7 (3.1)	42 (19.8)	49 (11.2)
	$40,000-$49,999	7 (3.1)	30 (14.2)	37 (8.4)
	$50,000-$59,999	9 (4.0)	26 (12.3)	35 (8.0)
	$60,000-$69,999	17 (7.5)	14 (6.6)	31 (7.1)
	$70,000-$99,999	23 (10.2)	14 (6.6)	37 (8.4)
	More than $99,999	100 (44.2)	10 (4.7)	110 (25.1)
**Health condition, n (%)**				
	Major	36 (15.9)	134 (63.2)	170 (38.8)
	Minor	190 (84.1)	78 (36.8)	268 (61.2)
**When had the condition, n (%)**				
	Past	114 (50.4)	63 (29.7)	177 (40.4)
	Current	112 (49.6)	149 (70.3)	261 (59.6)

## Results

### Psychometrics

The results suggest that the overall Information Scale, the overall Decision Making Scale, and all the subscales of these two scales were internally consistent and reliable (Cronbach alpha coefficients ranged from .95-.71 for the younger age group, and .98-.78 for the older age group); confirmatory factor analyses supported the construct validity of the HIWQ (see [42] for detailed descriptions of the reliability and construct validity of the instrument). Furthermore, the overall scores for both the Information and Decision Making Scales were significantly correlated with those for their corresponding global items (for Information, “How much information would you like to have about this condition?”; For Decision Making, “Who do you think should make the decision related to this specific health condition?”). Specifically, for young adults, the correlation was .42 (*P*<.01) for the information dimension and .34 (*P*<.01) for the decision-making dimension. For older adults, the correlation was .61 (*P*<.01) for the information dimension and .49 (*P*<.01) for the decision-making dimension. These significant correlations support the convergent validity of the HIWQ.

### Internet Use Frequency

Internet use frequency was measured by the following item: How often do you use the Internet? Responses ranged from Never (1) to Everyday (6). Significant difference was found between the younger and older age groups’ Internet use frequencies, with the younger age group having significantly more frequent Internet use than the older age group (younger age group mean 5.98, SD 0.33; older age group mean 3.50, SD 2.00; *t*_436_=17.42, *P*<.01).

### Relationship Between Internet Use Frequency and Overall Preferences

Results of multilevel modeling analysis (Tables 2 and 3) showed that, after controlling for age group, gender, general health status, health condition (major vs minor), whether the condition was current or past, how long the condition lasted, severity of the condition, how knowledgeable participants were about the condition, marital status, education level, ethnicity, income level, and Big Five personality, Internet use frequency was positively related to the overall preference rating (*γ*=.15, *P*<.05), suggesting that frequent Internet users preferred significantly more information and decision-making autonomy than did infrequent Internet users. Internet use frequency did not predict the random slope between rating dimension (information vs decision making) and the overall preference rating, suggesting that there was no interaction among Internet use frequency, overall information preference, and overall decision-making preference.

### Relationship Between Internet Use Frequency and Each Type of Preference

In the following analyses, the main effects of age group, gender, general health status, health condition, whether the condition was current or past, how long the condition lasted, severity of the condition, how knowledgeable participants were about the condition, marital status, education level, ethnicity, income level, and Big Five personality on preference ratings were controlled for. The results of multilevel modeling analysis for the relationship between Internet use frequency and each type of preference are also reported in Tables 2 and 3.

**Figure 1:**
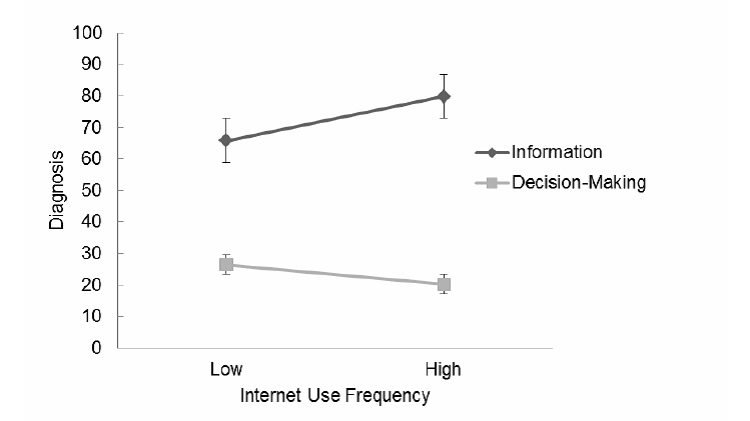
Interaction between Internet use frequency and rating dimension (Information vs Decision Making) for the Diagnosis Subscale.

For the diagnosis subscale, the main effect of Internet use frequency on preference rating was not significant. However, results of multilevel modeling analysis showed that Internet use frequency was positively related to the random slope between rating dimension (information vs decision making) and preference rating (γ=.57, *P*<.01), suggesting an interaction effect of Internet use frequency on this rating dimension. These results indicated that frequent Internet users preferred obtaining more information but less decision-making autonomy about diagnosis than did infrequent Internet users (Figure 1).

**Figure 2:**
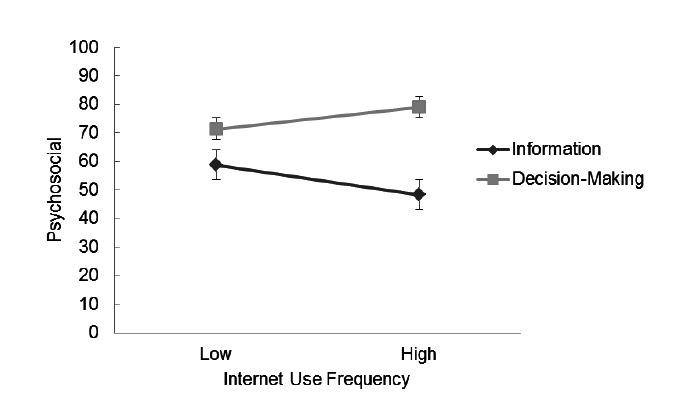
Interaction between Internet use frequency and rating dimension (Information vs Decision Making) for the Psychosocial Subscale.

For the psychosocial subscale, the main effect of Internet use frequency on preference rating was not significant. However, results of multilevel modeling analysis showed that Internet use frequency was negatively related to the random slope between rating dimension (information vs decision making) and preference rating (*γ*=-.51, *P*<.01), suggesting an interaction effect of Internet use frequency on this rating dimension. These results indicated that frequent Internet users preferred obtaining less information but more decision-making autonomy about psychosocial aspects than did infrequent Internet users (Figure 2).

**Figure 3:**
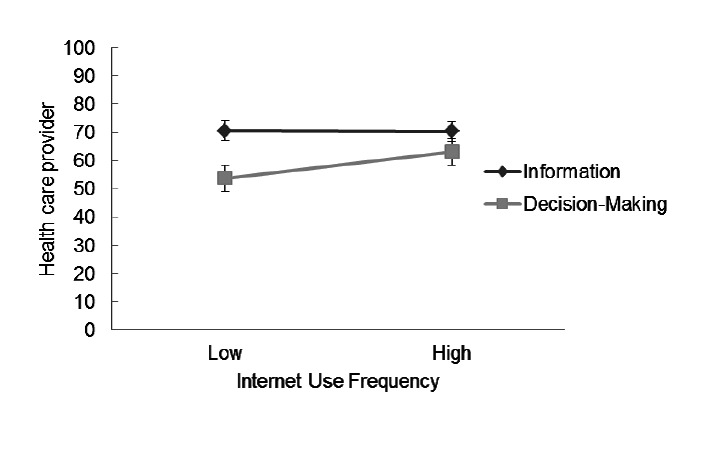
Interaction between Internet use frequency and rating dimension (Information vs Decision Making) for the Health Care Provider Subscale.

For the health care provider subscale, the main effect of Internet use frequency on preference rating was not significant. However, results of multilevel modeling analysis showed that Internet use frequency was negatively related to the random slope between rating dimension (information vs decision making) and preference rating (*γ*=-.27, *P*<.05), suggesting an interaction effect of Internet use frequency on this rating dimension. These results indicated that frequent Internet users preferred obtaining less information but more decision-making autonomy about health care providers than did infrequent Internet users (Figure 3).

Results of multilevel modeling analysis showed that Internet use frequency was positively related to preference rating for the laboratory test (*γ*=.15, *P*<.05), self-care (*γ*=.15, *P*<.05), and CAM (*γ*=.32, *P*<.01) subscales. For these subscales, Internet use frequency did not predict the random slope between rating dimension (information vs decision making) and preference rating. These results suggested that frequent Internet users would prefer obtaining more information and decision-making autonomy about laboratory testing, self-care, and CAM than infrequent Internet users would. For the treatment subscale, Internet use frequency was not significantly related to preference rating or the random slope between rating dimension and ratings.

**Table 2:** Multilevel modeling results – overall, diagnosis, treatment, and laboratory test (Level 2 [ie, between-person level] N=438; Level 1 [ie, within-person level] N=876; unstandardized coefficients are reported).

Variable		Overall	Diagnosis	Treatment	Laboratory test
**Random intercept (*β*_0_)**					
	Intercept (*γ*_00_)	5.68 ^a^	4.81 ^a^	5.69 ^a^	4.57 ^a^
	Age group (*γ*_01_)	.37	1.09 ^a^	.17	1.16 ^a^
	Gender (*γ*_02_)	-.20	-.21	-.22	.08
	Health condition (*γ*_03_)	-.15	-.29	-.29	-.20
	Condition time (*γ*_04_)	.00	.05	.21	.05
	Years of condition (*γ*_05_)	.01	.00	.01	.00
	Severity (*γ*_06_)	.06	.11	.16	.11
	Knowledgeable (*γ*_07_)	-.00	-.04	.08	.06
	General health status (*γ*_08_)	-.05	-.06	-.16	-.19
	Education (*γ*_09_)	-.02	.04	.03	-.02
	Single vs married (*γ*_010_)	.06	.25	.43	.25
	Separated vs married (*γ*_011_)	.09	.32	.73	-.32
	Divorced vs married (*γ*_012_)	.46	.29	.73 ^b^	.43
	Widowed vs married (*γ*_013_)	.10	.22	.31	-.05
	Living as married vs married (*γ*_014_)	.06	-.06	.83	-2.62 ^a^
	Asian vs white (*γ*_015_)	-.13	.09	-.26	.10
	African American vs white (*γ*_016_)	-.28 ^b^	-.11	-.03	-.07
	Latino vs white (*γ*_017_)	.18	-.08	-.56	.61
	Native American vs white (*γ*_018_)	-.91	-.15	.68	-.97 ^a^
	Pacific Islander vs white (*γ*_019_)	-.12	-.37	-1.18	.27
	Income (*γ*_020_)	-.02	-.01	-.02	-.07 ^b^
	Extraversion (*γ*_021_)	.01	.13 ^a^	.03	.04
	Agreeableness (*γ*_022_)	-.01	.01	.01	.00
	Conscientiousness (*γ*_023_)	.04	.03	-.02	.03
	Neuroticism (*γ*_024_)	.02	.11 ^b^	.09	.03
	Openness (*γ*_025_)	.01	-.01	.11	-.01
	Internet use frequency (*γ*_026_)	.15 ^b^	.11	.15	.15 ^b^
	Residual variance (*υ*_*1*_^2^)	.88	.69	.36 ^b^	1.07
**Random slope for preference dimension (*β*_1_)**					
	Intercept (*γ*_10_)	2.77 ^a^	4.95 ^a^	4.53 ^a^	4.98 ^a^
	Internet use frequency (*γ*_11_)	.02	.57 ^a^	.07	.06
	Residual variance (*υ*_*1*_^2^)	5.71 ^a^	8.58 ^a^	5.15 ^a^	6.91 ^a^
Level 1 residual variance (*σ* ^2^)		1.22 ^a^	2.38 ^a^	3.38 ^a^	2.98 ^a^

^a^ *P*<.01.

^b^ *P*<.05.

**Table 3:** Multilevel modeling results – self-care, CAM, psychosocial, and health care provider (Level 2 [ie, between-person level] N=438; Level 1 [ie, within-person level] N=876; unstandardized coefficients are reported).

Variable		Self-care	CAM	Psychosocial	Health care provider
**Random intercept (*β*_0_)**					
	Intercept (*γ*_00_)	6.45 ^b^	5.79 ^b^	6.44 ^b^	6.45 ^b^
	Age group (*γ*_01_)	-.71 ^a^	.62	-.99 ^b^	.10
	Gender (*γ*_02_)	-.18	-.24	-.24	-.72 ^b^
	Health condition (*γ*_03_)	-.12	-.18	-.01	-.09
	Condition time (*γ*_04_)	-.09	-.03	-.01	-.13
	Years of condition (*γ*_05_)	.02	.02	.02 ^a^	.02
	Severity (*γ*_06_)	.18 ^a^	.09	.07	.08
	Knowledgeable (*γ*_07_)	-.12	-.02	-.02	-.04
	General health status (*γ*_08_)	-.09	-.15	.06	.05
	Education (*γ*_09_)	-.03	-.01	.03	-.08
	Single vs married (*γ*_010_)	-.44	.01	-.45	-.03
	Separated vs married (*γ*_011_)	-.57	.59	.43	-.07
	Divorced vs married (*γ*_012_)	.19	.85 ^a^	.48	.55
	Widowed vs married (*γ*_013_)	-.40	.32	.14	-.05
	Living as married vs married (*γ*_014_)	-1.21 ^a^	2.19 ^b^	-.78	-1.78 ^b^
	Asian vs white (*γ*_015_)	-.29	-.25	-.31	-.07
	African American vs white (*γ*_016_)	-.48 ^a^	-.46 ^a^	-.33 ^a^	-.16
	Latino vs white (*γ*_017_)	.09	-.02	.08	.28
	Native American vs white (*γ*_018_)	-1.63	-.91	-1.32	-.88
	Pacific Islander vs white (*γ*_019_)	1.28	-.60	.63	-1.43
	Income (*γ*_020_)	-.02	-.03	.02	-.01
	Extraversion (*γ*_021_)	-.13 ^a^	-.01	-.05	-.01
	Agreeableness (*γ*_022_)	.01	.05	-.03	.07
	Conscientiousness (*γ*_023_)	.08	.10	.02	-.02
	Neuroticism (*γ*_024_)	-.15 ^a^	.04	-.10	.09
	Openness (*γ*_025_)	.05	.04	-.02	.04
	Internet use frequency (*γ*_026_)	.15 ^a^	.32^b^	-.04	.13
	Residual variance (*υ*_*1*_^2^)	.97	.93	1.67	1.57
**Random slope for preference dimension (*β*_1_)**					
	Intercept (*γ*_10_)	2.18^b^	2.21^b^	-2.16^b^	1.20^b^
	Internet use frequency (*γ*_11_)	-.04	.07	-.51^b^	-.27 ^a^
	Residual variance (*υ*_*1*_^2^)	7.34^b^	10.56^b^	14.42^b^	9.19^b^
Level 1 residual variance (*σ* ^2^)		γ	γ	2.46^b^	4.47^b^

^a^ *P*<.05.

^b^ *P*<.01.

## Discussion

### Principal Findings

Promoting patient-centered health care requires an understanding of patient preferences for obtaining health information and decision-making autonomy. Recent developments in information and communication technologies have introduced complications to the scope and extent of patient participation [58-60]. Some argue that the Internet is bringing fundamental changes to the medical profession [58,61]. Drawing upon Paul Starr’s framework of medical professionalism [62], Blumenthal [58] has argued that the Internet has enabled patients to challenge two particular core attributes contributing to the distinctive competence of medical professionals. First, the Internet creates unprecedented opportunities for the general public to access vast amounts of medical knowledge previously known only to medical professionals, thus challenging the *cognitive* attribute of the medical profession. This argument is supported by empirical studies showing a large number of health consumers obtaining health information from the Internet [63,64]. Second, by generating convenient access to information about the credentials and experiences of medical professionals [65], the Internet also enables the general public to make informed decisions about the track record of their physicians [27,66], thus challenging the *collegial* attribute of the medical profession (ie, self-monitoring and self-discipline within the profession itself) [58]. Ample empirical evidence supports this argument. For instance, through various online tools including social media sites, health consumers are actively describing, rating, and sharing their experiences of health care facilities and physicians, and on the basis of peer experience, making decisions regarding which facility or physician to go to [67-69]. In fact, this bottom-up approach has become so prevalent that medical professionals have begun to explore how to make best use of such patient-generated ratings and content [70,71].

The findings of the present study provide further empirical evidence for these arguments by revealing a positive correlation between Internet use and patient participation. Specifically, with regard to RQ1 (Is there a significant relationship between Internet use frequency and the overall preferences for obtaining health information and decision-making autonomy?), our findings show that Internet use frequency was positively related to overall preference rating, suggesting that frequent Internet users preferred significantly more information and decision-making autonomy than did infrequent Internet users. Interestingly, findings from this study (reported elsewhere) also suggest that age was not associated with overall preference rating [42]. Therefore, compared with age, Internet use frequency appears to be more strongly associated with overall preference for health information and decision-making autonomy in this study. These findings have important implications for medical practice: when medical professionals attempt to gauge how much information to provide to patients or try to decide how much they should involve patients in medical decision-making, they may be better off if they base their decisions on patients’ Internet use frequency rather than age per se.

With regard to RQ2 (Does the relationship between Internet use frequency and information and decision-making preferences differ with respect to seven different aspects of health conditions, ie, diagnosis, treatment, laboratory testing, self-care, CAM, psychosocial aspect, and health care providers?), our findings suggest that the relationship between Internet use frequency and different types of preferences varies. Specifically, compared with infrequent Internet users, frequent Internet users preferred *more* information but *less* decision-making autonomy for diagnosis, *more* information and *more* decision-making autonomy for laboratory testing, CAM, and self-care, and *less* information but *more* decision-making autonomy for the psychosocial and health care provider aspects. For treatment, we did not find a significant difference between frequent and infrequent Internet users in their information and decision-making preferences.

These findings challenge others widely reported in the literature. In particular, there seems to be a consensus that patients are interested in obtaining more information but are not as interested in participating in decision-making [25,44,72-75]. The context of such a “consensus” though, as we have explained, is the fact that previously only a very limited range of preferences was measured, while other types of preferences—that might not be perceived as important by medical professionals but nonetheless are important from the patient’s perspective—were largely ignored [17,42]. Using the HIWQ, which covers a broader range of preferences than previous instruments and presents parallel items on the information and decision-making scales, we have been able to develop a more comprehensive view of patient preferences consisting of nuances previously ignored.

These nuances have important implications for medical practice, particularly given the increasing emphasis on patient-centered health care [3]. For instance, our findings suggest that Internet use frequency is positively associated with overall preference for health information and participation in decision-making, but that when overall preference is broken down into different aspects, the relationship between Internet use frequency and different types of preferences varies from one aspect to another. Thus, to encourage patient participation, medical professionals might want to consider promoting different aspects of participation to different extents to better accommodate patients’ preferences. For instance, medical professionals might want to provide frequent Internet users with more information and more decision-making autonomy about laboratory testing, CAM, and self-care than they would provide to infrequent Internet users. However, medical professionals might not need to provide as much psychosocial information for frequent Internet users as for infrequent Internet users.

Previous research suggests that age is a strong predictor of patient preferences [16], with younger adults having a significantly stronger desire for both information and decision-making autonomy than their older counterparts [16,19,21,44-47]. However, our findings suggest that age was not associated with the overall preference rating or preference about treatment and CAM; furthermore, on the subscales where age was related to preference ratings (diagnosis, psychosocial aspect, health care providers, and self-care), its effect is in line with that of Internet use frequency [42]. These findings suggest that, just as when they make decisions regarding *overall* information and decision-making preference, medical professionals, when they try to decide how much of a specific type of information to provide to patients or how much to involve patients in specific types of decision-making, may want to base their decisions on patients’ Internet use frequency rather than age.

### Limitations and Future Directions

This study used a convenience sample. Considering that some of the relationships tested were statistically significant, size of the current sample did allow sufficient statistical power for testing the effects of interest. Still, the results may not be representative. Caution should be taken in generalizing the findings to the general population. The sample consisted of two groups, undergraduates 18-32 years old and older adults 50-100; these groups were frequent and infrequent Internet users, respectively. Additional research should address a broader range of Internet use frequency to determine whether these results could be replicated across groups with varying levels of Internet use frequency (and it would be especially interesting to compare and contrast older adults who are *frequent* Internet users with younger adults who are *infrequent* Internet users to better understand the relationships among age, Internet use frequency, and preference for participation). Furthermore, in this study we measured the construct of “Internet use frequency”, which is a subconstruct of “Internet use” that may involve broader variation than the “frequency” of use. It would be interesting in future research to further validate the findings in a population of patients seeking care whose interest in technology and actual use of it may vary more widely than the two populations (ie, older adults at a computer class and college students) examined in this study.

The HIWQ, when administered in cross-sectional studies like the present one, provides only a snapshot view of preferences. Yet, experiences of illness can span months or even years, and preferences for obtaining health information and decision-making autonomy may change over time [76-78]. In future research, it will be necessary to administer the HIWQ multiple times to assess and compare if and how patient preferences for participation might evolve over the course of their conditions. Another limitation is that some of the decision-making subscales showed lower Cronbach alpha values in the younger age group [42]. One possible reason is that the younger participants had less life experience with making important medical decisions. Therefore, the constructs and the items were less familiar to them, which might lead to lower Cronbach alphas. Future research should further investigate this issue by collecting data from other younger adult samples. Additionally, in our study, we had only one global item for the information scale and one item for the decision-making scale. Future research may use another measure with multiple items for each of these scales to provide more persuasive evidence for the instrument’s convergent validity. Finally, as reviewed above, patient preferences are often used in the literature as indicators of patient participation in their own health care. However, preferences may not already be a perfect proxy for actual participation. Further research would need to confirm correlation between preference and actual participation in health information seeking and decision making.

### Conclusions

Internet applications have created unprecedented opportunities for patient participation through improved access to a wide range of health information previously difficult for the general public to obtain [33,63-65]. Patients are now better equipped with knowledge necessary to make more informed decisions about a broad range of health care-related issues [27,28,34-39,66-69]. Not surprisingly, it has been suggested that the Internet is bringing fundamental changes to the medical profession [58,61], as patients become more informed, more participatory, and consequently, more empowered [79]. Our findings, while supporting this general argument about the relationship between Internet use frequency and patient participation and empowerment, also reveal novel nuances in this relationship (eg, when patient preference is broken down into seven aspects, the relationships between Internet use frequency and type of information preference and its corresponding decision-making preference clearly vary across those aspects).

Previous research suggests that age, gender, education, culture, the role of being a patient, severity of health condition, and personality can help explain the variance in patient preferences [16,19,21,44-50]. This study reveals a new related factor for patient preferences: Internet use frequency, which was significantly related to not only overall preference but also preferences for several types of information and decision-making autonomy. These findings may have important implications for medical practice. For example, medical professionals may want to take into account their patients’ Internet use frequency when understanding if, how much, and in what ways their patients might wish to participate in their own health care.

## Multimedia Appendix 1

HIWQ items.

## Abbreviations

CAM: complementary and alternative medicine
HIW: Health Information Wants
HIWQ: Health Information Wants Questionnaire
