# Supplementary material for: Internet Use Frequency and Patient-Centered Care: Measuring Patient Preferences for Participation Using the Health Information Wants Questionnaire
Source: J Med Internet Res. 2013 Jul 1;15(7):e132. doi: 10.2196/jmir.2615 (PMC3714005; doi:10.2196/jmir.2615)
Supplement: Supplementary file 1 [file jmir_v15i7e132_app1.pdf]

Health Questionnaire

Please fill out this questionnaire. All of your responses will be treated confidentially. Any published document regarding these responses will not identify individuals. Thank you in advance for your help!

Part 1

1. Please think about a specific health condition you had in the past or currently have, and **write down the name of this health condition here: ______________________________________**

1a. Is this a condition you had in the past or currently have? Please circle ONE:

$_1$Had in the past $_2$Currently have

1b. How long have you had this condition? _________Year(s) and _________ Month(s)

2. How severe do you think this health condition is/was? Please circle ONE:

1	2	3	4	5
Not severe at all	A little severe	Moderately severe	Very severe	Extremely severe

3. How knowledgeable do you think you are about this condition?

1	2	3	4	5
Not at all knowledgeable	A little knowledgeable	Moderately knowledgeable	Very knowledgeable	Extremely knowledgeable

4. How much information would you like to have about this condition?

1	2	3	4	5
None	A little	Some	Most	All

5. Circle the appropriate number of **each row** to indicate how much information you would like to have about each of the following areas **related to this specific health condition**:

**How much information would you like to have?**					
1. Information about the stage of this health condition (e.g., how advanced it is, how far it has spread)	None<br>1	A little<br>2	Some<br>3	Most<br>4	All<br>5
2. Information about how severe this health condition is	None<br>1	A little<br>2	Some<br>3	Most<br>4	All<br>5
3. Information explaining why further referral is necessary	None<br>1	A little<br>2	Some<br>3	Most<br>4	All<br>5
4. Information about whether this health condition is contagious	None<br>1	A little<br>2	Some<br>3	Most<br>4	All<br>5
5. Information explaining how a medication may help to treat this health condition	None<br>1	A little<br>2	Some<br>3	Most<br>4	All<br>5
6. Information about the specific drug(s) prescribed	None<br>1	A little<br>2	Some<br>3	Most<br>4	All<br>5
7. Information about changing medications	None<br>1	A little<br>2	Some<br>3	Most<br>4	All<br>5

Circle the appropriate number of **each row** to indicate how much information you would like to have about each of the following areas **related to this specific health condition**:

How much information would you like to have?					
8. Information about the benefits and risks of different laboratory tests	None 1	A little 2	Some 3	Most 4	All 5
9. Information about the procedures of laboratory tests	None 1	A little 2	Some 3	Most 4	All 5
10. Information about interpretations of the results of laboratory tests	None 1	A little 2	Some 3	Most 4	All 5
11. Information about how this health condition may affect my work	None 1	A little 2	Some 3	Most 4	All 5
12. Information about how this health condition may affect my personal life (e.g., sexual activity, smoking, alcohol use, hobbies)	None 1	A little 2	Some 3	Most 4	All 5
13. Information about how to care for a wound or incision at home	None 1	A little 2	Some 3	Most 4	All 5
14. Information about the benefits and risks of using complementary/alternative medicine alone versus in combination with conventional medicine	None 1	A little 2	Some 3	Most 4	All 5
15. Information about when to get complementary/alternative medicine	None 1	A little 2	Some 3	Most 4	All 5
16. Information about where to get complementary/alternative medicine	None 1	A little 2	Some 3	Most 4	All 5

Circle the appropriate number of **each row** to indicate how much information you would like to have about each of the following areas **related to this specific health condition**:

How much **information** would you like to have?					
17. Information about support groups where I can talk with other people in similar situations	None<br>1	A little<br>2	Some<br>3	Most<br>4	All<br>5
18. Information about how the treatment may affect feelings about myself	None<br>1	A little<br>2	Some<br>3	Most<br>4	All<br>5
19.Information about how to involve my family in dealing with feelings about this health condition	None<br>1	A little<br>2	Some<br>3	Most<br>4	All<br>5
20. Information about the credentials, experiences, or reputations of a particular medical facility	None<br>1	A little<br>2	Some<br>3	Most<br>4	All<br>5
21. Information about the credentials, experiences, or reputations of a particular medical specialist	None<br>1	A little<br>2	Some<br>3	Most<br>4	All<br>5

Part 2

1. Please write down here the name of the specific health condition that you wrote down on the first page: ______________________________, and continue thinking about this health condition while filling out the next section.

2. Who do you think should make the decision related to this specific health condition? **Circle** one of the following:

The doctor alone	Mostly the doctor	The doctor and myself equally	Mostly myself	Myself alone

3. Circle the appropriate cell of **<u>each row</u>** to indicate ***<u>who</u>* <u>you think should make the decision in each of the following areas</u>:**

Who do you think should make the decision?					
1. Decision regarding what stage of this condition it is (e.g., how advanced it is, how far it has spread)	The doctor alone	Mostly the doctor	The doctor and myself equally	Mostly myself	Myself alone
2. Decision regarding how severe this health condition is	The doctor alone	Mostly the doctor	The doctor and myself equally	Mostly myself	Myself alone
3. Decision regarding whether further referral is necessary	The doctor alone	Mostly the doctor	The doctor and myself equally	Mostly myself	Myself alone
4. Decision regarding whether this health condition is contagious	The doctor alone	Mostly the doctor	The doctor and myself equally	Mostly myself	Myself alone
5. Decision regarding whether to use a medication	The doctor alone	Mostly the doctor	The doctor and myself equally	Mostly myself	Myself alone
6. Decision regarding which specific drug(s) to use	The doctor alone	Mostly the doctor	The doctor and myself equally	Mostly myself	Myself alone
7. Decision regarding whether to change medications	The doctor alone	Mostly the doctor	The doctor and myself equally	Mostly myself	Myself alone

Circle the appropriate cell of **<u>each row</u>** to indicate ***<u>who</u>* <u>you think should make the decision in each of the following areas</u>:**

**Who do you think should make the decision?**					
8. Decision regarding what laboratory test(s) to use	The doctor alone	Mostly the doctor	The doctor and myself equally	Mostly myself	Myself alone
9. Decision regarding how to proceed with a given laboratory test	The doctor alone	Mostly the doctor	The doctor and myself equally	Mostly myself	Myself alone
10. Decision regarding how to interpret the results of a given laboratory test	The doctor alone	Mostly the doctor	The doctor and myself equally	Mostly myself	Myself alone
11. Decision regarding how to adapt to this health condition at work	The doctor alone	Mostly the doctor	The doctor and myself equally	Mostly myself	Myself alone
12. Decision regarding how to adapt to this health condition in my personal life	The doctor alone	Mostly the doctor	The doctor and myself equally	Mostly myself	Myself alone
13. Decision regarding how to care for a wound or incision at home	The doctor alone	Mostly the doctor	The doctor and myself equally	Mostly myself	Myself alone

Circle the appropriate cell of **<u>each row</u>** to indicate ***<u>who</u>* <u>you think should make the decision in each of the following areas</u>:**

**Who do you think should make the decision?**					
14. Decision regarding whether to use complementary/alternative medicine alone or in combination with standard medicine	The doctor alone	Mostly the doctor	The doctor and myself equally	Mostly myself	Myself alone
15. Decision regarding <u>when</u> to get complementary/alternative medicine	The doctor alone	Mostly the doctor	The doctor and myself equally	Mostly myself	Myself alone
16. Decision regarding <u>where</u> to get complementary/alternative medicine	The doctor alone	Mostly the doctor	The doctor and myself equally	Mostly myself	Myself alone
17. Decision regarding whether to join support groups to talk with other people in similar situations	The doctor alone	Mostly the doctor	The doctor and myself equally	Mostly myself	Myself alone
18. Decision regarding how to deal with feelings about myself as a result of the treatment	The doctor alone	Mostly the doctor	The doctor and myself equally	Mostly myself	Myself alone
19. Decision regarding how to involve my family in dealing with feelings about this health condition	The doctor alone	Mostly the doctor	The doctor and myself equally	Mostly myself	Myself alone
20. Decision regarding whether to go to a particular medical facility	The doctor alone	Mostly the doctor	The doctor and myself equally	Mostly myself	Myself alone
21. Decision regarding whether to see a particular medical specialist	The doctor alone	Mostly the doctor	The doctor and myself equally	Mostly myself	Myself alone
